# Anti-obesity effect of ethanolic extract from *Cosmos caudatus* Kunth leaf in lean rats fed a high fat diet

**DOI:** 10.1186/s12906-017-1640-4

**Published:** 2017-02-22

**Authors:** Hafeedza Abdul Rahman, Najla Gooda Sahib, Nazamid Saari, Faridah Abas, Amin Ismail, Muhammad Waseem Mumtaz, Azizah Abdul Hamid

**Affiliations:** 1 School of Chemical Sciences and Food Technology, Faculty of Science and Technology, Universiti Kebangsaan Malaysia, 43600 UKM Bangi, Selangor Malaysia; 2 Department of Food Science, Faculty of Food Science and Technology, Universiti Putra Malaysia, 43400 UPM Serdang, Selangor Malaysia; 3 Department of Nutrition and Health Sciences, Faculty of Medicine and Health Sciences, Universiti Putra Malaysia, 43400 Serdang, Selangor Malaysia; 4 Department of Chemistry, Faculty of Science, University of Gujrat, 50700 Gujrat, Pakistan; 5 Halal Products Research Institute, Universiti Putra Malaysia, Putra Infoport, 43400 UPM Serdang, Selangor Malaysia

**Keywords:** Anti-obesity, Herb, High fat diet, NMR, Rats

## Abstract

**Background:**

Obesity is a major health concern both in developed and developing countries. The use of herbal medicines became the subject of interest for the management of obesity due to its natural origin, cost effectiveness and minimal side effects. The present study aimed at investigating anti-obesity potential of ethanolic extract from *Cosmos caudatus* Kunth leaf (EECCL).

**Methods:**

In this study, the rats were randomly divided into six groups i.e., (1) Normal Diet (ND); (2) Normal Diet and 175 mg/kgBW of EECCL (ND + 175 mg/kgBW); (3) Normal Diet and 350 mg/kgBW of EECCL (ND + 350 mg/kgBW); (4) High Fat Diet (HFD); (5) High Fat Diet and 175 mg/kgBW of EECCL (HFD + 175 mg/kgBW); (6) High Fat Diet and 350 mg/kgBW of EECCL (HFD + 350 mg/kgBW). The anti-obesity potential was evaluated through analyses of changes in body weight, visceral fat weight, and blood biochemicals including total cholesterol, triglycerides, high-density lipoprotein cholesterol (HDL-c), low-density lipoprotein cholesterol (LDL-c), leptin, insulin, adiponectin, ghrelin and fecal fat content. In addition, metabolite profiling of EECCL was carried out using NMR spectroscopy.

**Results:**

Rats receiving EECCL together with HFD showed significant (*p* < 0.05) reduction in body weight gain compared to rats receiving HFD only. At the end of study, the body weight gain of EECCL treated rats was not significantly (*p* > 0.05) different with those of ND rats. Other related obesity biomarkers including plasma lipid profiles, insulin, leptin, ghrelin and adiponectin levels also showed significant improvement (*p* < 0.05). Administration of EECCL caused significant (*p* < 0.05) increase in fecal fat excretion, which validates the hypothesis of lipase inhibition, an anti-obesity mechanism similar to standard drug of Orlistat. The ^1^H-NMR spectra of EECCL ascertained the presence of catechin, quercetin, rutin, kaempherol and chlorogenic acid in the extract.

**Conclusion:**

Conclusively, EECCL showed anti-obesity properties by inhibition of intestinal lipid absorption and modulation of adipocytes markers.

## Background

Obesity is characterized by increase in the size and/or number of adipocytes in the adipose tissue [1]. Globally, it is estimated that over 205 million men and 297 million women were obese, which account for a total of more than 600 million adults worldwide [2]. Studies by the World Health Organization (WHO) indicated that at least 2.8 million people die each year as a result of being overweight or obese [2]. Obesity has now been considered as a major health concern both in developed and developing countries. It is also associated with various comorbidities including hyperlipidemia, diabetes, fatty liver, cancer and atherosclerosis [3–6]. Prevention of obesity is therefore very crucial, not only in adults but also in children.

There are many ways to prevent or control obesity, which includes, diet regimes, exercise and medication. However, the use of anti-obesity drugs such as Orlistat and Sibutramine has been reported to cause adverse side effects including high blood pressure, constipation, dry mouth, headache, heart attack and insomnia [7, 8]. Consequently, more trials have been conducted on the use of herbal medicines that were reported to possess anti-obesity potential in-vitro and in-vivo. These herbal medicines became the subject of interest due to its natural origin, cost effectiveness and minimal side effects [9].

*Cosmos caudatus* Kunth also known as ‘Ulam raja’ is a popular salad among Malaysians and has traditionally been used throughout the centuries for its nutritional and medicinal properties. Previously, the researchers have evaluated numerous medicinal effects of *Cosmos caudatus* Kunth both in vitro and in vivo, and it has been revealed that *Cosmos caudatus* extract play a preventive role against various degenerative diseases including hyperlipidemia, hypertension and diabetes. Perumal et al. [10] reported that, 4 weeks treatment of hyperlipidemic rats with *Cosmos caudatus* extract helped to effectively reduce their atherogenic index and glucose level, while Amalia et al. [11] found that *Cosmos caudatus* showed antihypertensive effect by decreased cardiac output and induction of diuresis. Loh et al. [12] revealed that *Cosmos caudatus* extract effectively inhibit α-amylase and α-glycosidase activity, key enzymes that control post-prandial hyperglycemia [13]. Human studies also showed that 8 weeks supplementation of *Cosmos caudatus* significantly improves insulin resistance and insulin sensitivity in type 2 diabetic patients [14].

The presence of quercetin, rutin and chlorogenic acid in *Cosmos caudatus* is well documented [15]. These compounds are known to take significant part in regulation of obesity [16–18]. Results from our previous study (results under publication) showed the ability of phenolic rich ethanolic extract of *Cosmos caudatus* Kunth leaves (EECCL) in inhibiting activities of fat metabolizing enzymes i.e., pancreatic lipase (PL) and lipoprotein lipase (LPL) in vitro. Thus suggesting that EECCL might be useful in the prevention and treatment of obesity by limiting dietary fat digestion, absorption and accumulation in adipose tissue. Animal models have provided major contributions to the investigations of various complex diseases including obesity [19]. They are very useful and widely used in obesity research as they readily gain weight and reached obesity in just few months of feeding with high fat diet. The greatest advantage of using animal models is that they allow strict control of all factors, which is very crucial in safety and efficacy study. In this study, male *Sprague dawley* rats were used with strict control of diet and environmental conditions to ensure reliability of data obtained at the end of the study. This model with its physiological properties replicates many of the features observed in obese human and also mimics human obesity better when compared to the genetic model [20, 21]. Therefore in the present study, the anti-obesity effect of EECCL was evaluated using lean *Sprague dawley* rats fed a high fat diet (HFD) with and without EECCL supplementation.

## Methods

### Materials

Standard rat chow (Gold Coin, Selangor, Malaysia), high fat diet (MP Diets, USA), tween 20, ethanol, biochemical kits for total cholesterol, triglyceride, LDL-c, and HDL-c were procured from Randox (Roche Diagnostics GmbH, Sandhofer Strasse, Mannheim), insulin, leptin, ghrelin and adiponectin levels were obtained from Mercodia Rat Insulin ELISA Kit, Uppsala, Sweden, RayBio Rat Leptin ELISA kit, Norcross, GA, USA, AssayMax Rat Adiponectin ELISA Kit, and RayBio Rat Ghrelin Enzyme Immunoassay Kit, Norcross, GA, USA. All the solvents, reagents and chemical used in the present study were of analytical grade.

### Plant material and extraction

The fresh leaves of *Cosmos caudatus* Kunth were collected from Agricultural Farm, Universiti Putra Malaysia (UPM) Selangor, Malaysia. A voucher specimen of *Cosmos caudatus* (H022) was deposited in the herbarium of Facuty of Forestry, Universiti Putra Malaysia. The leaves were immediately quenched using liquid nitrogen and lyophilized under pressure (−50 °C, 48–72 h, LABONCO, Labonco Corporation, Kansas City, Missouri, USA) until constant weight. The leaf extract of the plant was prepared using the modified method of Chang et al. [22]. The dried plant sample was ground using a commercial grinder, sieved, and stored at −80 °C until further use. *Cosmos caudatus* Kunth leaves were extracted with ethanol (100%). Dried plant material was soaked with ethanol (1:10) at room temperature for 72 h (collected every 24 h and pooled). The extracts were then filtered through Whatman No1 filter paper and solvent evaporated off using rotary evaporator at 40 °C. The resulting viscous substance (EECCL) was freeze dried to ensure complete removal of solvent and kept at −80 °C before feeding it to the rats.

### ^1^H-NMR analysis for metabolite profiling of EECCL

A modified method by Kim et al. [23] and Kim et al. [24] was used for the preparation of NMR sample. In 2 mL microcentrifuge tubes, 25 mg of EECCL was weighed and dissolved in 0.375 mL of methanol-*d*_*4*_ and 0.375 mL of KH_2_PO_4_ buffer in D_2_O (pH 6.0) containing 0.1% Trimethylsilypropionic acid sodium salt (TSP). The microcentrifuge tubes containing plant samples were then vortexed for 1 min at room temperature followed by ultrasonication for 15 min and centrifugation for 10 min at 13,000 rpm to separate the supernatant from \any insoluble components. The clear supernatant (0.6 mL) was transferred to NMR tubes and subjected to ^1^H-NMR analysis. The ^1^H-NMR analysis was performed using a 500 MHz Varian INOVA NMR spectrometer (Varian Inc., California, USA), operated at 499.887 MHz frequency and spectra were recorded at 26 °C. Each spectrum consisted of 64 scans, width of 20 ppm with 3.53 min of acquisition time. Chenomix software (v. 5.1. Alberta, Canada) was used to conduct phasing and baseline correction.

### Experimental animals and design

**Fig. 1:**
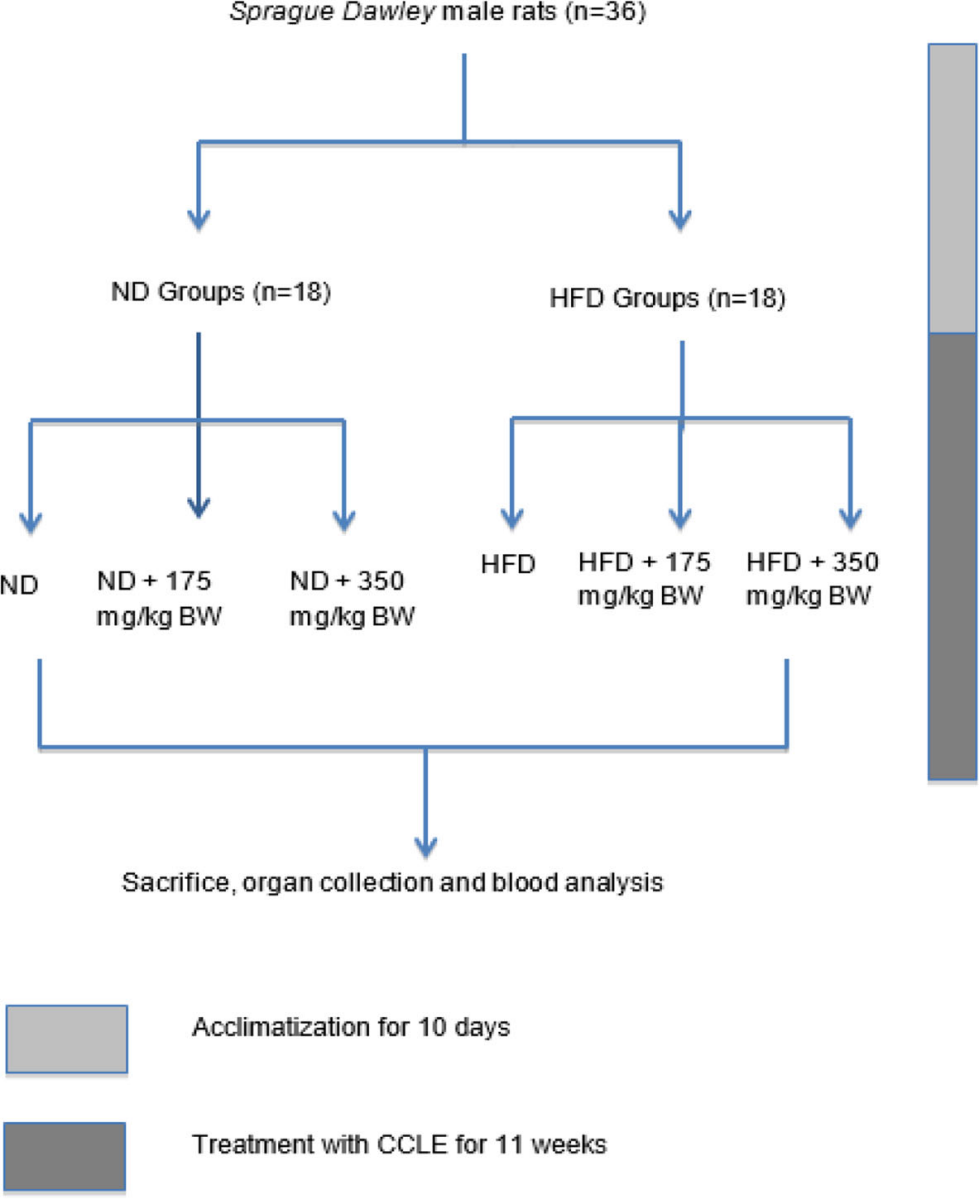
Experimental design for the determination of EECCL on prevention of obesity in lean rats fed a high fat diet. N: Normal diet, HFD: High fat diet, ND + 175 mg/kg: Normal diet + 175 mg/kg body weight of EECCL, ND + 350 mg/kg: Normal diet + 350 mg/kg body weight of EECCL, HFD + 175 mg/kg: High fat diet + 175 mg/kg body weight of EECCL, HFD + 350 mg/kg: High fat diet + 350 mg/kg body weight of EECCL, BW: Body weight

Thirty-six male *Sprague dawley* rats (5 to 6 weeks old) weighing at 185.46 ± 13.33 g (mean ± SD) (weight of rats on the day received from supplier) were purchased from A-Sapphire Enterprise Sdn. Bhd. (Kuala Lumpur, Malaysia). Rats were housed in a polycarbonate cages (15 × 25 cm) with stainless steel covers (2 rats in a cage with wood shavings as bedding) at 26–28 °C temperature under dark (12-h) and light (12-h) cycles with free access to standard animal chow/high fat diet and water *ad libitum*. During acclimatization period, the rats were given normal rat chow along with water *ad libitum.* After 10 days of adaptation to the environment, the rats were randomly divided into six groups (*n* = 6/group) as follows: (1) Normal Diet (ND); (2) Normal Diet and 175 mg/kgBW of EECCL (ND + 175 mg/kgBW); (3) Normal Diet and 350 mg/kgBW of EECCL (ND + 350 mg/kgBW); (4) High Fat Diet (HFD); (5) High Fat Diet and 175 mg/kgBW of EECCL (HFD + 175 mg/kgBW); (6) High Fat Diet and 350 mg/kgBW of EECCL (HFD + 350 mg/kgBW). The schematic experimental design is illustrated in Fig. 1. Rats in the normal diet and normal diet with extracts groups (group 1, 2 and 3) were given standard rat chow (Gold Coin, Selangor, Malaysia) whereas rats in group 4,5 and 6 were given high fat diet (MP Diets, USA). The composition of each diet is listed in the Table 1. Health conditions of all rats were monitored daily and no adverse events were observed throughout the study. At the beginning of the experiments the weights of all rats were recorded at 195.50 ± 12.61 g (mean ± SD) (weight of rats after 10 days of acclimatization). All experiments and biochemical analysis were conducted using 36 rats with triplicate measurements. The permission to conduct this study was obtained from ACUC (Animal Care and Use Committee), Faculty of Medicine and Health Sciences, UPM Malaysia (ACUC No: UPM/FPSK/PADS/BR-UUH/00463).

**Table 1:** Composition of normal and high fat diet used in the study

Normal Diet (ND)		High Fat Diet (HFD)	
Gold Coin, Malaysia (3.27 kcal/g)		MP Diet, USA (4.39 kcal/g)	
Ingredients	Percentage/Amount	Ingredients	Percentage/Amount
Crude Protein	21–23%	Casein Purified High Nitrogen	20.00%
Crude Fibre	5.00%	DL-Methionine	0.30%
Crude Fat	3.00%	Sucrose	30.58%
Moisture	3.00%	Corn Starch	20.00%
Calcium	0.8–1.2%	Coconut Oil Hydrogenated	20.00%
Phosphorus	0.8–1.2%	Alphacel, Non-Nutritive Bulk	5.00%
Nitrogen Free Extract	49.00%	DL-a-Tocopherol Powder (250 IU/gm)	0.12%
Vitamin A	10 MIU	AIN-76 Mineral Mix	4.00%
Vitamin D3	2.5 MIU	MP Vitamin Diet Fortification Mixture 1.2 X Normal Amount	
Vitamin E	15.0 g		

AIN-76 Mineral Mix contain the following ingredients (1000 g): Calcium Phosphate Dibasic 500.00 g, Sodium Chloride 74.00 g, Potassium Citrate Monohydrate 220.00 g, Potassium Sulfate 52.00 g, Magnesium Oxide 24.00 g, Manganese Carbonate (43–48% Mn) 3.50 g, Ferric Citrate (16–17% Fe) 6.00 g, Zinc Carbonate (70% ZnO) 1.60 g, Cupric Carbonate (53–55% Cu) 0.30 g, Potassium Iodate 0.01 g, Sodium Selenite 0.01 g, Chromium Potassium Sulfate 0.55 g, and Sucrose 118.00 g

### Administration of EECCL

Treatments were started on the 11th day, after 10 days of acclimatization. The dried crude EECCL was diluted with 5% (w/v) Tween 20 for complete solubility and administered daily according to the dosage for each group, (175 mg/kg or 350 mg/kgBW) for group 2,3,5, and 6 while rats in group 1 and 4 received 5% (w/v) Tween 20. They were given by oral gavage everyday using a force-feeding needle for 11 weeks.

### Determination of body weight gain, food intake and energy intake

Body weight change and food intake was measured according to method by Akase et al. [25]. Intake of food was measured weekly on a cage basis and expressed as g of food/day. Initial body weight of all animals was measured before they were fed with either ND or HFD for 11 weeks. At the end of each week, the weight gain (%) was calculated as followed:

$$ \mathrm{Weight}\ \mathrm{gain}\left(\%\right)=\frac{\mathrm{New}\ \mathrm{weight}\left({\mathrm{W}}_1\right)-\mathrm{Initial}\ \mathrm{weight}\left({\mathrm{W}}_0\right)}{\mathrm{Initial}\ \mathrm{weight}\left({\mathrm{W}}_0\right)}\times 100 $$

### Collection of plasma, liver, lung, kidney, heart, testis, visceral fats, and feces

Blood samples of overnight fasted rats were collected at the end of treatment (week 11) at 8 a.m in the morning by cardiac puncture under general anesthesia through intraperitoneal injection of ketamine and xylazine mixture (0.1 mL/100 g body weight of rat), which contains 90 mg/kg ketamine and 9 mg/kg xylaine. Ketamine and xylazine mixture was used according to the IACUC guidelines of anesthesia. The same combination of anesthesia was also used previously in anti-obesity study of germinated brown in high-fat diet induced rats [26]. The mixture also was used because it produce short-term surgical anesthesia with good analgesia. The collected blood was transferred into an EDTA tube following plasma separation by centrifugation at 3500 rpm under room temperature conditions for 15 min. The collected plasma was stored at −80 °C for further biochemical analysis. After 11 weeks of treatment, all the rats were sacrificed. The fats and organs like lung, liver, kidney, testis and heart were also weighed. Feces were collected in the middle and final week of the study for determination of fecal fat content. All experimental procedure were conducted in a specified surgical room of Animal House, Faculty of Medicine and Health Sciences, UPM Malaysia.

### Determination of fecal fat content

Fecal fat content of the rats were determined based on the slightly modified method described by [27, 28]. Briefly, feces (0.5 g) were soaked in 2 mL distilled water and homogenized completely. It was then stored at 4 °C for 24 h followed by homogenization by vortex for 1 min. Extraction of lipids from feces was executed using 7.5 mL chloroform : methanol (1:2, v:v) for 30 min, followed by the addition of 2.5 mL of chloroform and deionized water and shaking for another 30 min. Resultant mixtures were then centrifuged (at 2000 × g) for 15 min, the lipophilic layer was isolated and dried under vacuum.

### Biochemical measurements

Various biochemical parameters were measured including lipid profiles of plasma TG (triglyceride), TC (total cholesterol), LDL-c (low-density lipoprotein cholesterol) and HDL-c (high-density lipoprotein cholesterol) (Roche Diagnostics GmbH, Sandhofer Strasse, Mannheim), liver and kidney profile of alanine aminotransferase (ALT), alkaline phosphatase (ALP), aspartate aminotransferase (AST), gamma-glutamyl transferase (GGT), urea, creatinine, insulin (Mercodia Rat Insulin ELISA Kit, Uppsala, Sweden), leptin (RayBio Rat Leptin ELISA kit, Norcross, GA, USA, Cat# ELR-Leptin-001), adiponectin (AssayMax Rat Adiponectin ELISA Kit, Cat# ERA2500-1), ghrelin (RayBio Rat Ghrelin Enzyme Immunoassay Kit, Norcross, GA, USA, Cat# EIA-GHR-1). Concentration of LDL-c was calculated by Friedwald’s formula, whereas HDL-c was measured by a commercial direct non-precipitation method as per manufacturer instructions [29, 30]. All procedures were carried out according to manufacturer’s protocols.

### Statistical analysis of data

Data obtained was expressed as mean ± standard deviation (SD). The experimental data was analyzed by one-way analysis of variance (ANOVA) with Duncan’s post hoc test using SPSS Version 20.0. Moreover, *p* < 0.05 was considered to describe the significant difference.

## Results

### Metabolite identification of EECCL from ^1^H-NMR spectra

**Fig. 2:**
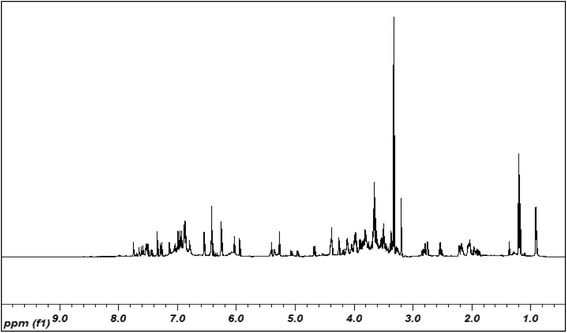
Representative of 500 MHz ^1^H-NMR spectra of EECCL from δ 0.50 to 10.0

**Fig. 3:**
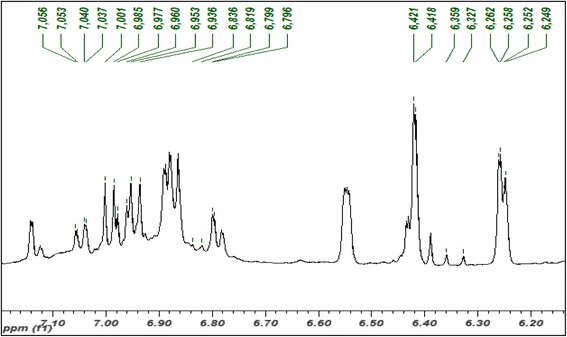
Expended 500 MHz ^1^H-NMR spectra of EECCL from δ 6.1 to 7.1. Values above the spectra indicate peak picking of 1H-NMR signals (ppm) of bioactive metabolites identified in EECCL

**Fig. 4:**
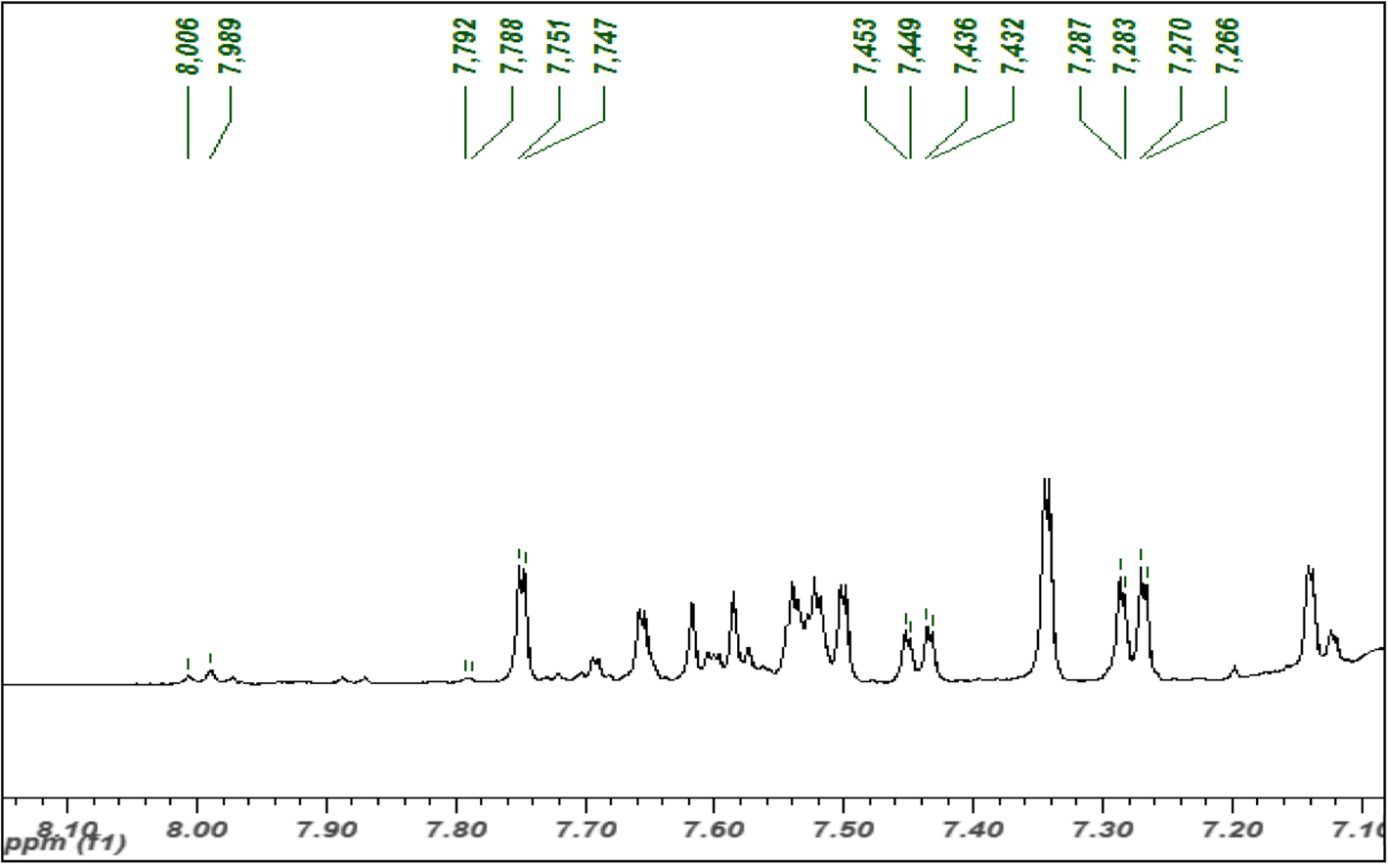
Expended 500 MHz ^1^H-NMR spectra of EECCL from δ 7.1 to 8.1. Values above the spectra indicate peak picking of 1H-NMR signals (ppm) of bioactive metabolites identified in EECCL

The full ^1^H-NMR spectra of EECCL is shown in Fig. 2, while the expanded aromatic regions are shown in Figs. 3 and 4. Trimethylsilypropionic acid sodium salt (TSP) was used as internal standard and for calibration of the NMR chemical shifts. The water suppression technique (pre-sat) was used to remove the undesired residual water signal. A typical ^1^H-NMR spectra of plant reveals signals of metabolites i.e., amino acids, organic acids and sugars in the aliphatic region (δ 0.5–3.0) and carbohydrate region (δ 3.0–5.5). The aromatic region (δ 5.5–9.0) describes various distinctive signals of secondary metabolites such as phenolics and flavonoids. All identified metabolites in EECCL are tabulated in Table 2.

**Table 2:** ^1^H-NMR chemical shifts (δ) and coupling constants (Hz) of metabolites identified in EECCL

Metabolite	^1^H-NMR signals
Quercetin	δ 6.25 (d, *J* = 1.5 Hz)
	δ 6.42 (d, *J* = 1.5 Hz)
	δ 6.80 (d, *J* = 1.5 Hz)
	δ 6.83 (d, *J* = 8.5 Hz)
	δ 6.98 (d, *J* = 8.5 Hz)
	δ 7.75 (d, *J* = 2.0 Hz)
	δ 7.79 (d, *J* = 2.0 Hz)
	δ 7.45 (dd, *J* = 8.5 Hz, 2.0 Hz)
Quercetin 3-*O*-α-rhamnoside	δ 6.25 (d, *J* = 1.5 Hz)
	δ 6.42 (d, *J* = 1.5 Hz)
	δ 6.80 (d, *J* = 1.5 Hz)
	δ 6.83 (d, *J* = 8.5 Hz)
	δ 7.29 (dd, *J* = 8.0 Hz, 2.0 Hz)
	δ 7.75 (d, *J* = 2.0 Hz)
	Anomeric proton rhamnosyl
	δ 4.54 (d, *J* = 2.0 Hz)
	Methyl signal;
	δ 0.92 (d, *J* = 6.0 Hz)
Quercetin 3-*O*-β-glucoside	δ 6.25 (d, *J* = 1.5 Hz)
	δ 6.42 (d, *J* = 1.5 Hz)
	δ 6.80 (d, *J* = 1.5 Hz)
	δ 6.83 (d, *J* = 8.5 Hz)
	δ 7.75 (d, *J* = 2.0 Hz)
	δ 5.08 (d, *J* = 7.5 Hz)
	Anomeric proton glucosyl
	δ 4.97 (d, *J* = 7.5 Hz)
Rutin	δ 6.25 (d, *J* = 1.5 Hz)
	δ 6.95 (d, *J* = 8.5 Hz)
	δ 7.54 (dd, *J* = 8.5 Hz, 2.5 Hz)
	Anomeric proton rhamnosyl
	δ 4.54 (d, *J* = 2.0 Hz)
	Anomeric proton glucosyl
	δ 4.97 (d, *J* = 7.5 Hz)
Chlorogenic acid	δ 2.07 (m)
	δ 2.22 (m)
	δ 7.06 (dd, *J* = 8.5, 1.5 Hz)
	δ 7.14 (d, *J* = 1.5 Hz)
	Signal for caffeoyl
	δ 6.36 (d, *J* = 16.0 Hz)
	δ 7.62 (d, *J* = 16.0 Hz)
	Signal for quinic
	δ 1.91 (d, *J* = 10.0 Hz)
Catechin	δ 2.56 (dd, *J* = 7.5 Hz, 16.0 Hz)
	δ 2.85 (m)
	δ 3.91 (m)
	δ 6.44 (d, *J* = 2.0 Hz)
Epicatechin	δ 6.44 (d, *J* = 2.0 Hz)
	δ 7.00 (d, *J* = 8.0 Hz)
	δ 7.29 (dd, *J* = 8.0 Hz, 2.0 Hz)
	δ 4.97 (d, *J* = 7.5 Hz)
Kaempferol	δ 6.26 (d, *J* = 2.0 Hz)
	δ 6.44 (d, *J* = 2.0 Hz)
	δ 6.80 (d, *J* = 1.5 Hz)
	δ 7.00 (d, *J* = 8.0 Hz)
	δ 8.01 (d, *J* = 8.5 Hz)
Sucrose	δ 4.19 (d, *J* = 8.5 Hz)
	δ 5.42 (d, *J* = 3.5 Hz)
β glucose	δ 4.60 (d, *J* = 8.0 Hz)
α glucose	δ 5.20 (d, *J* = 3.5 Hz)
Alanine	δ 1.50 (d, *J* = 7.5 Hz)
Valine	δ 1.02 (d, *J* = 7.0 Hz)
	δ 1.11 (d, *J* = 6.5 Hz)
	δ 2.22 (m)
Fatty acid	δ 1.35 (m)
Choline	δ 3.20 (s)
Isocitric acid	δ 4.12 (d, *J* = 5.0 Hz)

In general the NMR spectra of EECCL showed dominance in the carbohydrate and aromatic region. In the amino acids region, signal for alanine was revealed at δ 1.50 (d, *J* = 7.5 Hz), while signals for valine at δ 1.02 (d, *J* = 7.0 Hz), δ 1.11 (d, *J* = 6.5 Hz) and δ 2.22 (m). In the carbohydrate region, the anomeric proton signals of α-glucose δ 5.20 (d, *J* = 3.5 Hz), β-glucose δ 4.60 (d, *J* = 8.0 Hz), and sucrose δ 4.19 (d, *J* = 8.5 Hz), δ 5.42 (d, *J* = 3.5 Hz) were also detected.

The expansion of the aromatic region as shown in Fig. 3 further exposed signals of quercetin that were visualized at δ 6.25 (d, *J* = 1.5 Hz), δ 6.26 (d, *J* = 2.0 Hz), δ 6.42 (d, *J* = 1.5 Hz), δ 6.80 (d, *J* = 1.5 Hz), δ 6.83 (d, *J* = 8.5 Hz), δ 6.98 (d, *J* = 8.5 Hz), δ 7.75 (d, *J* = 2.0 Hz), δ 7.79 (d, *J* = 2.0 Hz) and δ 7.45 (dd, *J* = 8.5 Hz. 2.0 Hz). Quercetin 3-*O*-α-rhamnoside were also attributed due to the resonance signals at δ 7.29 (dd, *J* = 8.0 Hz, 2.0 Hz), δ 7.75 (d, *J* = 2.0 Hz) with a methyl signal from the rhamnosyl moiety attributed at δ 0.92 (d, *J* = 6.0 Hz). Quercetin 3-*O*-β-glucoside were attributed with the signals at 6.25 (d, *J* = 1.5 Hz), and δ 7.75 (d, *J* = 2.0 Hz) while the signal for the anomeric proton of glucosyl moieties was found at δ 5.08 (d, *J* = 7.5 Hz).

**Fig. 5:**
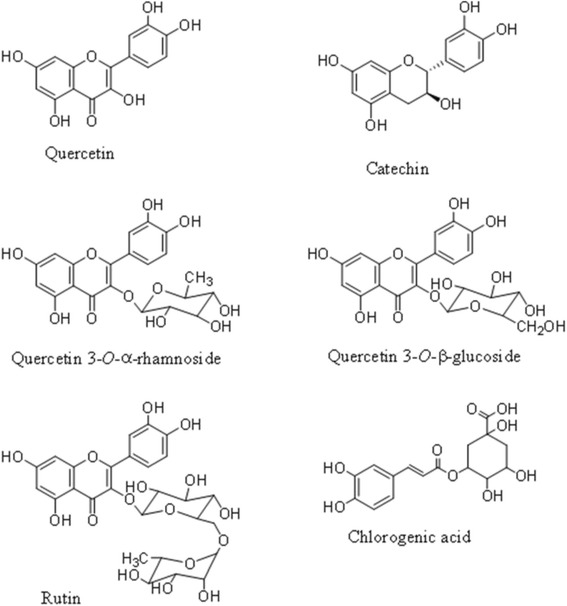
Structure of major compounds found in EECCL

Signals for chlorogenic acid was characterized at δ 2.07 (m), δ 2.22 (m), δ 7.06 (dd, *J* = 8.5, 1.5 Hz), δ 7.14 (d, *J* = 1.5 Hz). Furthermore, chlorogenic acid of the caffeoyl moiety showed signals at δ 6.36 (d, *J* = 16.0 Hz) and δ 7.62 (d, *J* = 16.0 Hz), while quinic moiety showed signal at δ 1.91 (d, *J* = 10.0 Hz). The characteristic signals of rutin were observed at δ 6.25 (d, *J* = 1.5 Hz), δ 6.95 (d, *J* = 8.5 Hz) and δ 7.54 (dd, *J* = 8.5 Hz, 2.5 Hz) with anomeric proton glucosyl at δ 4.54 (d, *J* = 2.0 Hz) and rhamnosyl at δ 4.97 (d, *J* = 7.5 Hz). The signals for catechin were detected at δ 2.56 (dd, *J* = 7.5 Hz, 16.0 Hz), δ 2.85 (m), δ 3.91 (m) and δ 6.44 (d, *J* = 2.0 Hz). Structures of potential bioactive compounds found in EECCL are presented in Fig. 5.

### Effects of EECCL on body weight gain, visceral fat mass, food and energy intake of rats

**Fig. 6:**
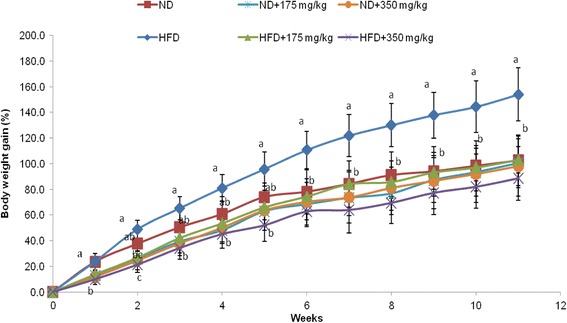
The effects of EECCL on percent body weight gain in rats for 11 weeks of treatment. Values are expressed as means ± SD (*n* = 6). Different letters (^a, b, c^) indicate significant difference (*p* < 0.05) between different groups as shown by ANOVA using SPSS Version 20. Body weight gain (%) = [(New weight–Initial weight)/Initial weight × 100]. N: Normal diet, HFD: High fat diet, ND + 175 mg/kg: Normal diet + 175 mg/kg body weight of EECCL, ND + 350 mg/kg: Normal diet + 350 mg/kg body weight of EECCL, HFD + 175 mg/kg: High fat diet + 175 mg/kg body weight of EECCL, HFD + 350 mg/kg: High fat diet + 350 mg/kg body weight of EECCL, BW: Body weight

The food intake and body weights of each rat were recorded on a weekly basis. Both the low (175 mg/kgBW) and higher dose (350 mg/kgBW) were used to see if the lower dose could have the same or better effect as the higher dose. At the end of the study (week 11), the body weight gain of the rats fed the HFD was 154.0%, whereas rats fed with the ND only gain 102.6% weight. The percentage of body weight gain was higher (1.50-fold) in the HFD group comparative to that of ND group (Fig. 6).

**Fig. 7:**
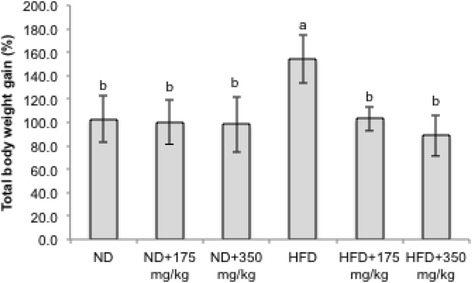
The effects of EECCL on total percent body weight gain in rats after 11 weeks of treatment. Values are expressed as means ± SD (*n* = 6). Different letters (^a, b^) indicate significant difference (*p* < 0.05) between different groups as shown by ANOVA using SPSS Version 20. Total percentage of body weight gain = [(Weight at week 11–Weight at week 0)/Weight at week 0 × 100%]. N: Normal diet, HFD: High fat diet, ND + 175 mg/kg: Normal diet + 175 mg/kg body weight of EECCL, ND + 350 mg/kg: Normal diet + 350 mg/kg body weight of EECCL, HFD + 175 mg/kg: High fat diet + 175 mg/kg body weight of EECCL, HFD + 350 mg/kg: High fat diet + 350 mg/kg body weight of EECCL, BW: Body weight

The % changes in body weight gain were given in relation to the initial weight at week 0. The percentage body weight gain of rats fed the HFD supplemented with both low and high dose of 175 mg/kgBW and 350 mg/kgBW of EECCL was significantly lower comparative to rats fed with the HFD alone for the entire period of study. EECCL with both low (HFD + 175 mg/kgBW) and high (HFD + 350 mg/kgBW) dosage prevented the weight gain by 32.99 and 42.47%, respectively when compared with the HFD group. Distinct separation can be seen from week 6 onwards, whereby all the 4 groups that received the extracts revealed non-significant (*p* > 0.05) difference with that of the ND group. The lowest gain in total body weight was observed for the HFD + 350 mg/kgBW group followed by the ND + 350 mg/kgBW, ND + 175 mg/kgBW and HFD + 175 mg/kgBW at 88.6, 98.2, 100.4 and 103.2%, respectively (Fig. 7).

**Fig. 8:**
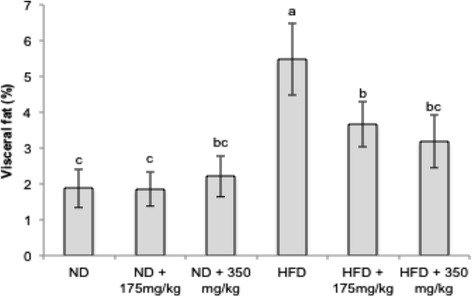
The effects of EECCL on percentage of visceral fats in rats for 11 weeks of treatment. Values are expressed as means ± SD (*n* = 6). Different letters (^a, b, c^) indicate significant difference (*p* < 0.05) between different groups as shown by ANOVA using SPSS Version 20. Fat (%) = [(Weight of fats/Weight of rats) × 100%]. N: Normal diet, HFD: High fat diet, ND + 175 mg/kg: Normal diet + 175 mg/kg body weight of EECCL, ND + 350 mg/kg: Normal diet + 350 mg/kg body weight of EECCL, HFD + 175 mg/kg: High fat diet + 175 mg/kg body weight of EECCL, HFD + 350 mg/kg: High fat diet + 350 mg/kg body weight of EECCL, BW: Body weight

Results also showed that the rats fed HFD for 11 weeks had significantly higher (5.48 ± 1.01%) visceral adipose tissues than that fed the ND (1.88 ± 0.55%). In the HFD groups, both low and high dosage of EECCL significantly decreased the visceral fats weight by 33.03 and 41.97% when compared to that of HFD group (Fig. 8). High dosage of EECCL in the HFD treated group showed better effect compared to the lower dose group although they were not-significantly different.

**Fig. 9:**
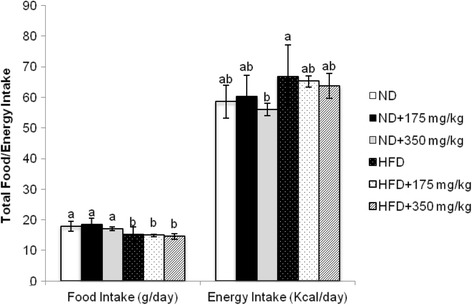
The effects of EECCL on food and energy intake in rats for 11 weeks of treatment. Values are expressed as means ± SD (*n* = 6). Different letters (^a, b^) indicate significant difference (*p* < 0.05) between different groups as shown by ANOVA using SPSS Version 20. Energy intake = Weight of food (g) × Total calorie of ND or HFD. ND = 3.27 kcal/g, HFD = 4.39 kcal/g. N: Normal diet, HFD: High fat diet, ND + 175 mg/kg: Normal diet + 175 mg/kg body weight of EECCL, ND + 350 mg/kg: Normal diet + 350 mg/kg body weight of EECCL, HFD + 175 mg/kg: High fat diet + 175 mg/kg body weight of EECCL, HFD + 350 mg/kg: High fat diet + 350 mg/kg body weight of EECCL, BW: Body weight

The food intake of the rats was significantly higher in ND and ND supplemented (with EECCL) groups in comparison to that received HFD (Fig. 9). However, the food intake of rats in the ND and HFD supplemented groups were not significantly different with their respective control groups. Although there was significant difference in the food intake (g/day) among the HFD and ND groups, energy intake (kcal/day) was similar in all HFD and ND groups except between the ND + 350 and HFD group (Fig. 9). Each gram of ND and HFD used in this study provides 3.27 and 4.39 kcal, respectively. The HFD group consumed 14.03% higher caloric intake comparative to the ND group. Results revealed that EECCL induced adipose weight loss and reduction in body weight gain without affecting the food or caloric intake.

### Effects of EECCL on organ weights of rats

Results showed that the weight of organs including liver, kidney, heart, lung and testis did not differ between all groups tested (Table 3).

**Table 3:** Organ weights of rats treated with EECCL for 11 weeks

	Dietary group					
	ND	ND + 175 mg/kgBW	ND + 350 mg/kgBW	HFD	HFD + 175 mg/kgBW	HFD + 350 mg/kgBW
Liver (g)	8.66 ± 0.82^a^	8.05 ± 0.42^a^	8.74 ± 1.35^a^	10.4 ± 2.09^a^	9.38 ± 1.05^a^	8.34 ± 0.53^a^
Kidney (g)	2.22 ± 0.21^a^	2.09 ± 0.33^a^	2.07 ± 0.15^a^	2.19 ± 0.28^a^	2.09 ± 0.23^a^	2.09 ± 0.26^a^
Heart (g)	1.25 ± 0.17^a^	1.16 ± 0.2^a^	1.16 ± 0.21^a^	1.20 ± 0.13^a^	1.29 ± 0.18^a^	1.17 ± 0.08^a^
Lung (g)	2.00 ± 0.27^a^	1.79 ± 0.26^a^	1.65 ± 0.34^a^	1.57 ± 0.08^a^	1.82 ± 0.28^a^	1.60 ± 0.28^a^
Testis (g)	1.55 ± 0.23^a^	1.68 ± 0.28^a^	1.53 ± 0.19^a^	1.54 ± 0.22^a^	1.40 ± 0.45^a^	1.59 ± 0.21^a^

Values are expressed as means ± SD (*n* = 6). Different letters indicate significant difference (*p* < 0.05) between different groups as shown by ANOVA using SPSS Version 20. *N* Normal diet, *HFD* High fat diet, *ND + 175 mg/kg* Normal diet + 175 mg/kg body weight of EECCL, *ND + 350 mg/kg* Normal diet + 350 mg/kg body weight of EECCL, *HFD + 175 mg/kg* High fat diet + 175 mg/kg body weight of EECCL, *HFD + 350 mg/kg* High fat diet + 350 mg/kg body weight of EECCL, *BW* Body weight

### Effects of EECCL on fecal fat content of rats

**Fig. 10:**
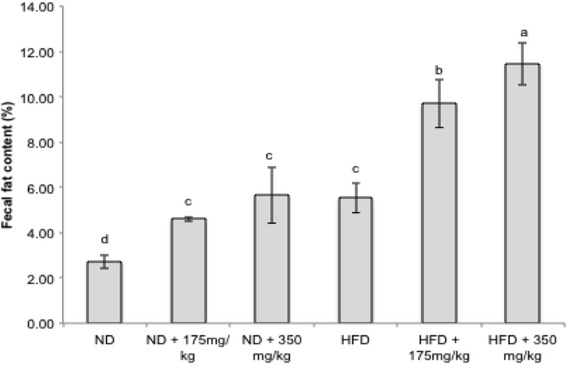
The effects of EECCL on fecal fat content in rats for 11 weeks of treatment. Values are expressed as means ± SD (*n* = 6). Different letters (^a, b, c, d^) indicate significant difference (*p* < 0.05) between different groups as shown by ANOVA using SPSS Version 20. N: Normal diet, HFD: High fat diet, ND + 175 mg/kg: Normal diet + 175 mg/kg body weight of EECCL, ND + 350 mg/kg: Normal diet + 350 mg/kg body weight of EECCL, HFD + 175 mg/kg: High fat diet + 175 mg/kg body weight of EECCL, HFD + 350 mg/kg: High fat diet + 350 mg/kg body weight of EECCL, BW: Body weight

Fecal fat content of all rats were analyzed at the end of treatment with EECCL (Fig. 10). Results of the study showed that fecal fat content of rats on HFD groups (5.54 ± 0.66%, 9.69 ± 1.05% and 11.46 ± 0.93% feces in HFD, HFD + 175 and HFD + 350 mg/kgBW, respectively) was significantly increased comparative to the ND group (2.72 ± 0.31% feces). Treatment with EECCL in HFD rats resulted in significant increase of fecal fat levels, the higher dose showed more prominent result (2.07-fold increased) than that of the lower dose (1.75-fold increased) of the extracts. All the groups (control and treated) did not suffer from diarrhea or other visible side effects. These results suggested that the treatment of EECCL prevented dietary fat absorption in the rats.

### Effects of EECCL on plasma lipid profiles of rats

Plasma TG levels of HFD-fed rats were increased by 69.6% compared to that of ND-fed rats (1.15 ± 0.20 mmol/L and 0.35 ± 0.02 mmol/L, respectively) (Table 4). However, no significant effect was observed in both the ND supplemented groups comparative to the ND group. Interestingly, both low and high dose supplementation of EECCL in the HFD treated group resulted in significantly lower plasma triglycerides levels. The HFD + 350 mg/kgBW group was found to be more potent in triglyceride lowering effect, although not significantly different with that of the lower dose of HFD + 175 mg/kgBW treated rats. After the completion of experimental period, HFD group showed 69.6% higher TG and 18.4% lower HDL-c levels when compared to that of ND group. Interestingly, both HFD treated groups exhibited cholesterol lowering effects, the higher dose of EECCL showed significant reduction in both TC and LDL-c levels (18.8 and 38.5% reduction, respectively) comparative to the HFD group. The high dose of EECCL showed stronger lowering effects on the levels of plasma TC and LDL-c than the lower dose of extract.

**Table 4:** The effects of EECCL on plasma obesity biomarkers level, liver and kidney functions in obese rats after 11 weeks of treatment

	Dietary group					
	ND	ND + 175 mg/kg	ND + 350 mg/kg	HFD	HFD + 175 mg/kg	HFD + 350 mg/kg
TG	0.35 ± 0.02^c^	0.32 ± 0.01^c^	0.41 ± 0.08^c^	1.15 ± 0.20^a^	0.70 ± 0.12^b^	0.62 ± 0.09^b^
TC	1.27 ± 0.17^a^	1.09 ± 0.22^ab^	1.26 ± 0.14^a^	1.28 ± 0.05^a^	1.14 ± 0.06^ab^	1.04 ± 0.05^b^
HDL-c	0.87 ± 0.14^ab^	0.86 ± 0.11^ab^	0.89 ± 0.08^a^	0.71 ± 0.04^b^	0.72 ± 0.05^b^	0.73 ± 0.03^ab^
LDL-c	0.25 ± 0.03^a^	0.25 ± 0.05^a^	0.27 ± 0.07^a^	0.26 ± 0.04^a^	0.24 ± 0.04^a^	0.16 ± 0.02^b^
Insulin	0.21 ± 0.03^c^	0.19 ± 0.02^c^	0.18 ± 0.02^c^	0.35 ± 0.03^a^	0.27 ± 0.06^b^	0.23 ± 0.04^bc^
Leptin	0.83 ± 0.20^bc^	0.65 ± 0.23^c^	0.68 ± 0.15^c^	2.05 ± 0.38^a^	1.33 ± 0.26^b^	1.12 ± 0.20^bc^
Adiponectin	17.19 ± 0.95^a^	17.00 ± 2.18^a^	16.11 ± 3.54^a^	10.26 ± 1.92^b^	17.49 ± 2.36^a^	17.07 ± 5.86^a^
Ghrelin	64.68 ± 8.64^ab^	78.92 ± 26.32^a^	54.78 ± 34.99^ab^	35.25 ± 8.64^b^	89.44 ± 25.94^a^	50.80 ± 7.37^ab^
AST	93.38 ± 24.24^a^	93.50 ± 11.25^a^	84.85 ± 12.09^a^	156.98 ± 113.32^a^	123.63 ± 32.56^a^	158.90 ± 48.09^a^
ALT	34.25 ± 4.38^bc^	33.25 ± 6.80^c^	32.08 ± 1.14^c^	51.90 ± 2.46^a^	56.38 ± 7.24^a^	45.95 ± 7.51^ab^
ALP	80.75 ± 6.02^bc^	79.75 ± 7.59^bc^	66.00 ± 16.85^c^	129.50 ± 23.70^a^	116.30 ± 26.14^ab^	94.00 ± 11.17^abc^
GGT	1.00 ± 0.00^b^	1.00 ± 0.00^b^	1.25 ± 0.50^b^	3.25 ± 1.26^a^	1.50 ± 1.00^b^	0.75 ± 0.50^b^
Urea	5.60 ± 0.35^a^	5.58 ± 0.33^a^	5.88 ± 1.07^a^	5.65 ± 0.90^a^	5.08 ± 0.49^a^	5.08 ± 0.54^a^
Creatinine	48.25 ± 0.96^a^	47.00 ± 3.16^a^	47.75 ± 5.19^a^	52.75 ± 3.30^a^	51.75 ± 2.06^a^	47.25 ± 2.06^a^

Values are expressed as means ± SD (*n* = 6). Different letters (^a, b, c^) indicate significant difference (*p* < 0.05) between different groups as shown by ANOVA using SPSS Version 20. *N* Normal diet, *HFD* High fat diet, *ND + 175 mg/kg* Normal diet + 175 mg/kg body weight *C. caudatus* extract, *ND + 350 mg/kg* Normal diet + 350 mg/kg body weight of EECCL, *HFD + 175 mg/kg* High fat diet + 175 mg/kg body weight of EECCL, *HFD + 350 mg/kg* High fat diet + 350 mg/kg body weight of EECCL, *TG* Total triglyceride, *TC* Total cholesterol, *HDL-c* High density lipoprotein–cholesterol, *LDL-c* Low density lipoprotein–cholesterol, *AST* Aspartate aminotransferase, *ALT* Alanine aminotransferase, *ALP* Alkaline phosphatase, *GGT* Gamma-glutamyl transferase. TG, TC, HDL-c and LDL-c were measured in mmol/L. Insulin was measured in μg/mL whereas leptin, adiponectin and ghrelin were measured in ng/mL. AST, ALT, ALP and GGT were measured in U/L whereas Urea and Creatinine were measured in mmol/L

### Effects of EECCL on plasma insulin, leptin, adiponectin and ghrelin levels of rats

The levels of plasma insulin, leptin, adiponectin and ghrelin were measured after 11 weeks of treatment with EECCL to determine the mechanism by which the extracts caused a significant reduction in the body weight gain of the rats (Table 4). Feeding both low (175 mg/kgBW) and high (350 mg/kgBW) dose of EECCL (in HFD rats) after 11 weeks resulted in significant (*p* < 0.05) decrease in the level of insulin i.e., 0.27 ± 0.06 μg/L (23.05%) and 0.23 ± 0.04 μg/L (33.14%) comparative to HFD group (0.35 ± 0.03 μg/L). At the end of study, non-significant difference was observed in the level of insulin between the HFD + 350 mg/kgBW EECCL treated HFD-fed rats and ND rats.

At the end of the study, HFD rats (2.05 ± 0.38 ng/mL) exhibited significantly increased plasma leptin levels compared to that of ND-fed rats (0.83 ± 0.20 ng/mL) of about 2.46-fold. Interestingly, in the HFD groups, low dose treatment showed ability to reduce the leptin level up to 34.96% while the high dose showed stronger effect of lowering leptin concentration up to 45.38% when compared with that of HFD group.

In investigating further the mode of action by which EECCL decreased excessive insulin concentrations and body weight gain in treated rats, plasma adiponectin concentrations were measured. At the completion of the study, ND rats (17.19 ± 0.95 ng/mL) exhibited significantly higher plasma adiponectin levels compared to the levels observed in untreated obese HFD-fed rats (10.26 ± 1.92 ng/mL) by about 67.59%. Treatment of HFD-fed rats with EECCL increased plasma adiponectin concentrations significantly beyond those in HFD. All the treated groups differ non-significantly with that of ND group.

The levels of plasma ghrelin were also determined in this study. Results showed that the ND (64.68 ± 7.72 ng/mL), ND-treated groups (78.92 ± 26.32 ng/mL and 54.78 ± 34.99 ng/mL) and HFD-treated groups (89.44 ± 25.94 ng/mL and 50.80 ± 7.37 ng/mL) showed higher level of plasma ghrelin concentrations compared to that (35.25 ± 2.36 ng/mL) of HFD rats.

### Effects of EECCL extract on kidney and liver function test of rats

The kidney and liver function tests were performed to determine if there were any toxic effects resulting from oral administration or treatment with EECCL. This assessment is very important in determining the safety of EECCL for further application. Results showed that both lower and higher dose of EECCL significantly inhibited the HFD induced increase in GGT levels by 53.8 and 76.9%, respectively and was not significantly different with that of ND and ND treated groups (Table 4). The levels of AST, ALT, ALP, urea and creatinine changed non-significantly in HFD treated groups comparative to the HFD-fed group. Similar results were seen in the ND treated rats when compared to the ND-fed group. It was depicted that administration of both low and high dose of the EECCL in the ND and HFD treated groups for 11 weeks did not induce any detectable adverse toxic effects in the rats studied.

## Discussions

The present study describes the NMR based metabolite profiling and anti-obesity potential of EECCL in HFD-fed lean rats. Identification of the metabolites of EECCL was based on comparison of the NMR chemical shifts and coupling constants with that of other studies or samples [31–33], which were measured under similar conditions. In general, the ^1^H-NMR spectra of ethanolic EECCL showed the presence of catechin, quercetin, rutin, kaempherol and chlorogenic acid, which were similar to those reported by Perumal et al. [10] and Mediani et al. [33].

Results from preliminary study on *Cosmos caudatus* Kunth extracted with different concentration of ethanol and water (100:0, 80:20, 60:40, 50:50 and 40:60) showed that *Cosmos caudatus* Kunth extracted with 100% ethanol (EECCL) exhibited best pancreatic lipase, antioxidant activity and highest phenolic and flavonoid content [34]. The different extracting solvent used in preparation of *Cosmos caudatus* Kunth extracts were important for the recovery of phenolic and flavonoid compounds and it was found that 100% ethanol (EECCL) was the most efficient solvent for extracting those compounds. Strong positive correlation between phenolic and flavonoid compounds and that of free radical scavenging and anti-lipase activity were observed, which suggested that these compounds were mainly responsible to the antioxidant and anti-obesity potential observed. Therefore the effect of phenolic rich EECCL extract on preventing obesity was further explored in this study using Sprague dawley rat model.

The current study ascertained the anti-obesity potential of EECCL in HFD-fed lean rats. The supplementation of HFD-fed rats with EECCL at 175 and 350 mg/kg levels significantly decreased body weight gain comparative to untreated HFD-fed rats without affecting food intake or energy intake. The suppression of body weight gain was accompanied with significant decreases in visceral fat mass among the HFD-treated groups. However, EECCL did not cause any significant suppression in body weight as well as their visceral fat mass in the ND treated rats comparative to the ND group. Supplementation of EECCL has little effect in normal rats comparable to the previous studies [25, 35].

Inhibition in the PL activity and augmentation of lipolysis are being considered to be the effective ways in management of body weight [36]. Present study revealed that, limitation in the absorption of lipid in the intestine is the potential mechanism by which the EECCL prevented weight gain in the HFD-fed rats. Previous studies also supported this hypothesis [37, 38]. Therefore, in this study, the ability of EECCL to increase the excretion of fecal fat and consequently fecal fat energy excretion partially explained the observed significant reduction regarding body weight gain of the treated rats.

Various in vitro and in vivo studies have revealed the presence of hypolipidemic compounds in EECCL [39–41]. Therefore, it is possible that the presence of these bioactive metabolites influences lipid dynamics and further prevents the treated rats from developing obesity. Effects of EECCL on lowering the TG, TC and LDL-c are consistent with that of other studies [42–44]. There might be two possible mechanisms behind the observed hypolipidemic effect of the extract, i.e., decrease in dietary cholesterol absorption in the intestinal tract or interference in the synthesis of cholesterol. The inhibition in the absorption of dietary fats usually limits the excess energy required for the storage of fats in adipose tissue, which was seen with the significant suppression of visceral fats in the treated rats. Study by Osada et al. [45] on apple phenols in rats showed that the antiatherogenic and hypolipidemic effects are associated with the inhibition of cholesterol absorption in the intestines of the rats and promotion of cholesterol catabolism. Hence, it could be depicted that the improvement in the lipid profiles were partially due to the phytochemical contents of EECCL.

Leptin, insulin, adiponectin and ghrelin are hormones involved in energy homeostasis and neuroendocrine regulation of appetite and satiety. It is known that adipose tissue does not only function as energy storing cells but also serve as a site for the secretion of various adipocytokines including leptin, adiponectin, resistin and others [46, 47]. Normally plasma leptin and insulin concentrations correlate positively while ghrelin and adiponectin correlate negatively with general adiposity and increase of fat mass [48]. Improving glucose and fat metabolism by normalization of these obesity related marker’s level is therefore a useful strategy in the treatment of obesity.

Obesity is associated with leptin and insulin resistance leading to hyperinsulinemia and hyperleptinemia, which are further linked with excessive body weight, especially central obesity [49, 50]. Therefore, improvement in glucose and fat metabolism by enhancement of both the insulin and leptin sensitivity and decreasing their levels is considered to be emphatic treatment strategy for obese patients. In the present study, plasma insulin level decreased in a dose dependent manner with a significant reduction in the level of insulin at 23.05 and 33.14% compared to that of HFD group. Prolong feeding of HFD has been reported to increase the insulin level, causing insulin resistance and hyperinsulinemia in rats [51]. It was revealed that in the present study, treatment with EECCL suppressed increase in insulin level in HFD fed rats.

Leptin and ghrelin contribute in the regulation of the feed intake and energy expenditure [49]. Feeding HFD has also been reported to increase leptin concentrations and cause leptin resistance in rats [52]. Kim et al. [53] and Lee et al. [54] reported that treatment with *Coix lachrymajobi var. mayeun* (seed) and *Diospyros kaki* (leaf) extracts exhibited 36 and 11% reduction in body weight gain of HFD-fed *Sprague dawley* rats through modulation of leptin. In this study, plasma leptin levels in the low and high dose treated groups was found to decrease by 34.96 and 45.38%, respectively in accordance with the decrease in visceral fat mass and suppression of body weight. This is probably due to the induced leptin resistance in the control rats. Insulin has been well recognized to play a role in determining leptin level [55]. Therefore, the significant decrease in the plasma leptin levels observed in the present study may have resulted in the suppression of body weight gain, visceral fat mass and plasma insulin concentration.

Adiponectin is one of the adipocytokines secreted by adipocytes. In addition, it has also been revealed that hypoadiponectemia is closely linked with insulin resistance along with hyperinsulinemia [56]. Various studies have demonstrated anti-atherogenic and anti-diabetic properties of adiponectin [57, 58]. In a previous study, green tea polyphenol (EGCG) significantly increased the level of adiponectin concentration in rats, which act as biomarker in obesity and its related complications [59]. A weight loss study revealed that adiponectin level was decreased in obesity, whereas increased with weight loss [60]. Similar effects have also been observed in the present study, whereby treatment of polyphenolic rich EECCL increases concentration of plasma adiponectin by 45.03 and 41.58% in both low and high dose treated groups compared to that of HFD. The significant increase of adiponectin concentration was in accordance with the decrease in visceral fat mass, body weight gain and plasma insulin concentrations. The results were in accordance with that of previous studies, which suggested that increase in adiponectin level is associated with weight loss [61].

Ghrelin has been recognized to influence feeding behavior, energy homeostasis and also gastrointestinal functions [62]. Various reports showed that body weight loss was accompanied with the increase in concentration of ghrelin [63, 64]. Our results showed that treatment with EECCL not only increases adiponectin levels, but also that of ghrelin levels. The observation that EECCL supplementation to the obese rats resulted in the increase of plasma ghrelin and adiponectin were consistent with the results of Hsu et al. [65] that reported the effects of green tea extracts in increasing the concentration of ghrelin and adiponectin in obese women.

The observed effects of EECCL on lipid profiles, percentage of visceral fats, hormones related to obesity, fecal fat excretion and suppression of body weight gain in our study were in compliance with the results reported by Nukitrangsan et al. [66] and Kishino et al. [67]. Study by Nukitrangsan et al. [66] showed that *Peucedanum japonicum Thunb* intake significantly prevented body weight gain, reduced abdominal fat, serum TG, leptin and increased fecal fat excretion in mice fed with HFD. Treatment with mixture consisted of *Salacia reticulate* extract and cyclodextrin also significantly suppressed body weight gain, plasma leptin, visceral fat mass and TG levels of rats fed with HFD [67].

Researchers have reported the presence of quercetin, catechin, proanthocyanidin, epicatechin, rutin, myricetin, naringenin, vitamin C and chlorogenic acid (crypto-chlorogenic acid, neo-chlorogenic acid, chlorogenic acid) in *Cosmos caudatus* leaves [10, 33, 68]. Quercetin has been reported to exhibit antioxidant and anti-obesity effects in animal studies [69, 70]. In addition, quercetin is also known to inhibit adipogenesis and apoptosis by activating monophosphate-activated protein kinase (AMPK) signal pathway in 3T3-L1 preadipocytes [71]. In another study, chlorogenic acid significantly reduced body weight, visceral fat mass, triglycerides, cholesterol, plasma leptin and insulin levels, whereas increased plasma adiponectin level, suggesting the multiple effects of chlorogenic acid in improving body weight, lipid metabolism and obesity related hormone levels in in obese mice [72]. It is likely that these bioactive components may influence the fat, lipid profiles and obesity related hormones dynamics and further prevents the treated rats from developing obesity.

As the metabolic patterns in rats are very much similar to that of human beings, employing this model using rats is rational in examining the ultimate impact of EECCL in preventing body weight. Even though rodents such as rats and mice are described as predominant model reflecting human obesity, there are still few physiological differences exist between rats and human such as absence of gall bladder, and vomit reflex in rats. Furthermore, the controlled environment and condition of rats in the study is not analogous to the human situation, whereby rats are housed in small cages that restrict physical activity and social interaction. Besides, the food and water also were accessible *ad libitum*. This situation does not normally happen in human. This study measure the effect of EECCL in preventing body weight gain in lean rats fed a HFD. Although positive effects can be seen in the results, the effects of the same extract on obese rats have not been evaluated. Therefore future study should be conducted to evaluate the ability of EECCL in reducing weight of obese rats model. Additional group such as physically active and physically active with EECCL treatment group should be included to evaluate not only the direct effects of EECCL but also combination effects of EECCL and active lifestyle on obese rats model.

Global epidemic of obesity is now considered as the leading cause of morbidity and mortality across the world. The prevalence of this disorder needed to be reversed and further prevented for protection of future generations. Despite the short term benefits of drug treatment in obesity, it also often associated with rebound in weight and negatife side effects [73]. Only few drugs have been registered for the treatment of obesity, which include orlistat (Xenical), dexfenfluramine (Redux), and rimonabant (Acomplia). However due to side effects and safety reason, only Orlistat were approved for long-term treatment of obesity [74]. To date, pharmacological treatments do not appear to be effective in producing sustained long-term weight loss [75, 76]. As research on obesity and the use of pharmaceutical drugs in management of obesity is highly controversial and often does not provide effective long-term solution, the role of medicinal herbs for prevention and amelioration of obesity has gained much interest.

In this study, EECCL showed to be able to significantly prevent weight gain even at lower dose and that the weight gain was not different compared to the rats fed with ND. It is also interesting to see that there was no indication of diarrhea or other abnormal discomfort or bowel activities in the treated rats, suggesting that EECCL has lesser side effects as opposed to the standard drug of Orlistat, whereby diarrhea and abdominal discomfort has been reported previously [77]. Most importantly, supplementation of EECCL at both dosages did not affect liver and kidney functions, indicating that the dosages used not only effective but also safe for the treatment. This results was supported by acute oral toxicity study on ethanolic extract of *Cosmos caudatus* that showed no visible signs of toxicity or death up to 5000 mg/kg body weight for 14 days of study [78]. In addition, histopathological observations in both acute and subacute toxicity studies also showed no detectable inflammation on the gross examination of internal organs without any necrosis, fatty infiltration, or alteration in cell structures [78]. However, further comprehensive toxicity studies should be conducted to ascertain the lack of chronic toxicity effects of EECCL intake in vivo.

## Conclusion

The present study revealed that EECCL was effective in preventing the increase in body weight gain, visceral fat mass, plasma TC, TG, LDL-c, insulin and leptin levels. Moreover, treatment with EECCL resulted in significant increase of ghrelin, adiponectin and fecal fat output in lean rats fed a HFD. Kidney and liver function test showed no signs of toxicity induced by 11 weeks treatment of EECCL on rats. The observed anti-obesity effects of EECCL in HFD-fed rats are likely to be caused by complex mixture of bioactive compounds (catechin, chlorogenic acid, epicatechin, kaempferol, rutin and quercetin derivatives) and modulation of obesity biomarkers measured. Results of this study highlights the basis for future investigations of EECCL as a source of natural product that has the potential to be developed as medicinal ingredients for prevention and treatment of obesity and other metabolic diseases in human.

## Abbreviations

%: Percent
^1^H-NMR: Proton Nuclear Magnetic Resonance
ACUC: Animal Care and Use Committee
ALP: Alkaline phosphatase
ALT: Alanine aminotransferase
AMPK: Monophosphate-activated protein kinase
ANOVA: Analysis of variance
AST: Aaspartate Aminotransferase
BW: Body weight
d: Doublet
dd: Doublet of Doublets
EDTA: Ethylenediaminetetraacetic Acid
EECCL: Ethanolic Extract of *Cosmos caudatus* Kunth Leaf
EGCG: Epigallocatechin Gallate
GGT: Gamma-glutamyl transferase
HDL-c: High Density Lipoprotein Cholesterol
HFD: High fat diet
J: Coupling Constant in Hz
LDL-c: Low Density Lipoprotein Cholesterol
LPL: Lipoprotein lipase
m: Multiplet
ND: Normal Diet
PL: Pancreatic lipase
rpm: Revolution per minute
s: Singlet
SPSS: Statistical Package for Social Science
TC: Total cholesterol
TG: Triglyceride
TSP: Trimethylsilanepropionic Acid Sodium Salt
U/L: Units per Liter
δ: Chemical Shift in ppm
